# Association Between Peripheral Blood Levels of Vitamin A and Autism Spectrum Disorder in Children: A Meta-Analysis

**DOI:** 10.3389/fpsyt.2021.742937

**Published:** 2021-09-30

**Authors:** Ni Wang, Yuqian Zhao, Junwei Gao

**Affiliations:** ^1^Nursing Office of Beijing Road Medical District, General Hospital of Xinjiang Military Region, Wulumuqi, China; ^2^Independent Researcher, Cangzhou, China; ^3^Department of Developmental Neuropsychology, School of Psychology, Third Military Medical University (Army Medical University), Chongqing, China

**Keywords:** vitamin A, autism spectrum disorder, peripheral blood, serum, association

## Abstract

Vitamin A is an essential fat-soluble micronutrient that plays important roles in a surprisingly wide variety of biological processes from early growth and development to brain maintenance. Numerous clinical studies have been conducted to explore the relationship between peripheral vitamin A levels and autism spectrum disorder (ASD), but the results of these studies are controversial. Therefore, we assessed the association between peripheral vitamin A levels and ASD in the present meta-analysis. Relevant records were retrieved through the Embase, Web of Knowledge and PubMed databases up to 13 November 2020. Reference lists were also searched and analyzed. Hedges' g with its corresponding 95% confidence interval (CI) was used to assess the association between peripheral vitamin A levels and ASD. A fixed or random effects model was selected according to a heterogeneity test in overall and subgroup analyses. Five records (six studies) with 935 ASD children and 516 healthy children were included in the present study. Significantly decreased peripheral vitamin A concentrations were observed in ASD children compared with healthy children (Hedges' g = −0.600, 95% CI −1.153 to −0.048, *P* = 0.033). A similar result was also obtained after removing the studies identified by Galbraith plots. In addition, no obvious publication bias was found in the meta-analysis. The findings of our meta-analysis suggested decreased peripheral vitamin A levels in ASD children compared with healthy children. Further investigations into the effects of vitamin A on the development of ASD are warranted.

## Introduction

Autism spectrum disorder (ASD) is a group of neurodevelopmental disorders characterized by impaired social communication and interaction, together with restricted, repetitive patterns of behavior, interests, or activities as described in *the fifth edition of the Diagnostic and Statistical Manual of Mental Disorders (DSM-5)*. The current estimated prevalence of ASD is ~2.7% in children and adolescents in the United States (1). ASD is becoming increasingly common worldwide and seriously reduces functioning in social relationships and the quality of life throughout an individual's lifespan (2, 3). Despite extensive basic and clinical investigations during the past several decades, research in this field is hampered by the limited understanding of the exact pathogenesis of ASD. It is generally believed that ASD is a multifactorial disorder that involves the interaction of genetic and environmental factors (4, 5).

Nutritional psychiatry has recently become a hot area of investigation due to the relatively low adverse effect profiles of nutritional products (6). An accumulating body of research indicates that early childhood nutritional status may regulate the development of the nervous system by affecting specific neuronal circuits, mitochondrial function, oxidative stress and neuroinflammation and potentially lead to ASD (7–11). Extensive animal studies have revealed that some micronutrients, such as vitamin D, vitamin B6 and docosahexaenoic acid (DHA) are related to autistic behaviors (12–14). In addition, abnormal trace element profiles and metabolic alterations have been reported in a series of clinical studies (15–17). However, few microelements were translated into effective markers of ASD. Therefore, we should try to define more nutritional markers to distinguish high-risk individuals with ASD.

Vitamin A (retinol), an essential lipophilic micronutrient, is involved in a complex signaling pathway that regulates gene expression in the central nervous system (CNS) and controls neuronal differentiation and neural tube patterning (18). Recently, considerable clinical evidence has suggested that abnormal peripheral vitamin A levels are associated with attention deficit hyperactivity disorder (ADHD), schizophrenia and generalized anxiety disorder (19–21). Moreover, vitamin A deficiency could lead to autism-like behaviors in a rat model (22). A clinical study indicated that maternal supplementation with micronutrients, including vitamin A, during pregnancy could reduce autism behaviors in offspring (23). Therefore, an accumulating body of research has tried to verify the relationship between peripheral vitamin A levels and ASD, but the results have been inconsistent. In 2020, a meta-analysis including four studies with 476 individuals revealed no significant association between peripheral vitamin A and ASD (24). In fact, more studies performed in 2020 with larger sample sizes sought to identify the association between peripheral vitamin A and ASD. Thus, we performed the present meta-analysis to confirm whether peripheral vitamin A levels were associated with ASD.

## Methods

### Publication Search

All eligible records published up to up to November 13, 2020 were retrieved by searching the PubMed, Web of Science, and Embase databases with no language restrictions. The following combination of terms was employed: “autism,” “autistic,” or “ASD”; and “vitamin A” or “retinol.” All retrieved records and their reference lists were screened carefully to further identify available articles. The search process was performed by two independent investigators.

### Inclusion and Exclusion Criteria

The inclusion criteria were as follows: (1) studies assessing the levels of vitamin A in peripheral blood of autistic patients; (2) the concentration of vitamin A in peripheral blood is reported; and (3) clinical studies. The exclusion criteria were as follows: (1) studies without sufficient data, such as the concentration of vitamin A; (2) case reports, reviews, comments, or abstracts; (3) *in vitro* studies; and (4) studies performed using animal models. For studies with overlapping data, only the study with the largest sample size was included. When the study reported a *P*-value as an inequality rather than an exact value, we would try our best to contact related authors to obtain the exact values for compatibility with meta-analysis software.

### Data Extraction

Retrieved records were screened according to their titles, abstracts and full texts. Data extraction was performed independently by two evaluators to avoid personal errors. Information on the first author, publication year, country, diagnostic criteria, source of ASD patients, vitamin A detection method, sample type, age and gender of individuals were extracted into a predesigned Excel sheet. Sample sizes, mean vitamin A concentrations, standard deviations (SDs), and *P*-values were also extracted as primary outcomes to generate effect sizes (ESs). When discrepancies occurred during in the process of data extraction, a third person was consulted to resolve these issues.

### Quality Assessment

The methodological quality of studies was assessed by two independent authors according to the Newcastle–Ottawa Scale (NOS). Eight items were included to assess the quality of studies. Disagreements were resolved by discussion with a third author.

### Statistical Analysis

In our study, most of the effect sizes (standardized mean differences, SMDs) were calculated by sample sizes, mean vitamin A concentrations, and corresponding SDs; the remaining effect size was calculated based on sample sizes and *P*-values. Then, the SMDs were converted to Hedges' g, which serves as a more unbiased measurement (25). The pooled Hedges' g and its corresponding 95% confidence interval (CI) were used to assess the strength of the association between vitamin A in peripheral blood and ASD. A fixed-effects model or random-effects model was selected according to the heterogeneity analysis results, which were assessed by the *I*^2^ value and a chi-square-based Q-test. When obvious heterogeneity existed among studies, a random-effects model was used to evaluate the relationship between peripheral vitamin A and ASD; in contrast, a fixed-effects model was used (26). Due to the limited number of included studies, we did not conduct sensitivity analysis. Publication bias was first visually evaluated by funnel plots generated by plotting the effect sizes against the precision (inverse of SE) for each study. Then, Egger's test was conducted to examine the publication bias quantitatively. The source of heterogeneity was first identified through subgroup analyses according to sample type and ethnicity. Given that there were fewer than three studies, subgroup analyses of plasma and whole blood were not performed. Then, Galbraith plots were drawn to further identify the source of heterogeneity across studies. All statistical analyses were conducted using Comprehensive Meta-analysis software (version 2; Biostat Inc) and STATA (version 15.0; STATA Corporation). *P* < 0.05 was considered statistically significant in our study.

## Results

### Study Characteristics

**Figure 1:**
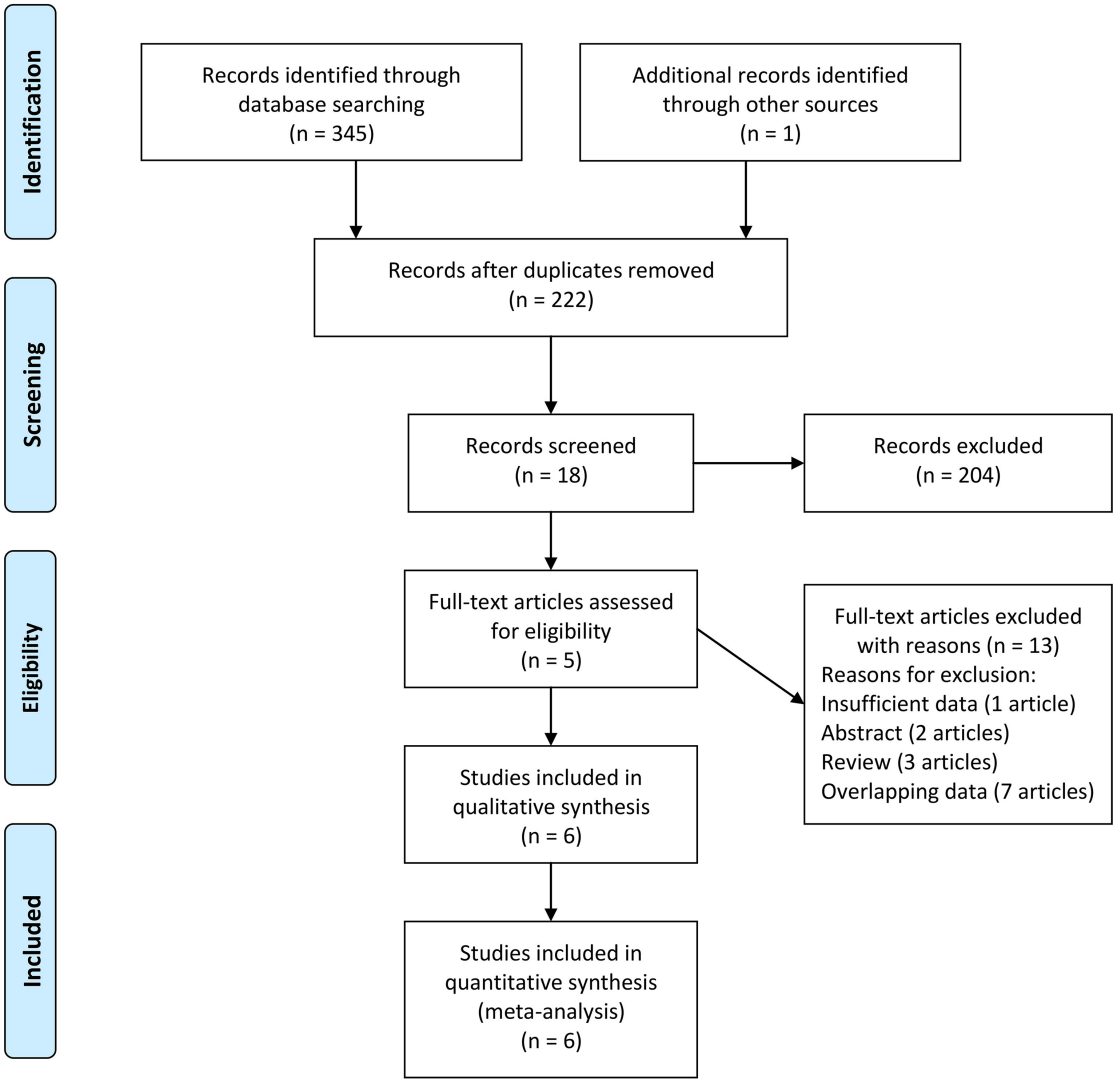
Flow diagram of study identification.

We first conducted a systematic search, which identified 71 records from PubMed, 166 records from Embase, 108 records from Web of Science, and one additional record identified through other sources. After removing 124 duplications and 204 unrelated records based on review of titles or abstracts, eighteen records underwent further screening through full-text reading. Furthermore, thirteen of the remaining records were excluded through full-text reading (7 records with overlapping data, 1 record with insufficient data, 2 reviews, and 3 abstracts). The study conducted by Zhu et al. reported the results in two different regions (27). We treated the report by Zhu et al. as two different studies: Zhu 2020 (1) and Zhu 2020 (2). Finally, five records (6 studies) with 1,451 unique participants were included in our meta-analysis to evaluate the association between the concentration of vitamin A and ASD (27–31). The flow diagram is shown in Figure 1.

Of the six studies included, four measured vitamin A levels in serum, one in plasma and one in peripheral whole blood. Three included studies were performed in China and three were performed in 2020. The levels of vitamin A in serum were determined using the high-performance liquid chromatography method. The subjects of the included studies were children. In most included studies, the diagnosis of ASD was based on *the Diagnostic and Statistical Manual of Mental Disorders (DSM)*. The characteristics of the included studies are presented in Table 1.

**Table 1:** Characteristics of the studies included in the meta-analysis.

**Sample**	**References**	**Country**	**Diagnostic criteria**	**Source**	**Detection method**	**Sample size**		**Cases**				**Controls**			**Methodological quality**
						**Case**	**Control**	**Age (years)**	**Gender (M/F)**	**Mean ± SD**	**Unit**	**Age (years)**	**Gender (M/F)**	**Mean ± SD**	
Serum	Zhu et al. (27) (1)^c^	China	DSM-5	The Children's Hospital of Chongqing Medical University and special education school of Chongqing, China	The high-performance liquid chromatography method	445	201	4.39 ± 1.25	379/66	224.87 ± 76.08^a^	μg/L	4.44 ± 0.82	102/99	243.52 ± 55.72^a^	7
Serum	Sweetman et al. (31)	Ireland	DSM-IV	The Sligo and South Leitrim area of the NW of Ireland	The high-performance liquid chromatography method	74	72	9.99 ± 4.2	65/9	350.56 ± 82.6	μg/L	6.43 ± 4.0	40/32	319.23 ± 82.8	5
Serum	Sun et al. (30)^c^	China	DSM-IV	The Children Development and Behavior Research Center of Harbin Medical University (Harbin, China)	The high-performance liquid chromatography method	53	53	4.94 ± 0.62	45/8	280 ± 59	μg/ L	4.95 ± 0.63	48/5	410 ± 78	7
Whole blood	Priya and Geetha (29)^c^	India	Check of Autism in Toddlers	A special school called V-excel Educational Trust at Chennai, Tamil Nadu, India	The UV spectrophotometric method	15	45	4–1^b^	NA	348 ± 52	μg/L	4–12	36/9	500 ± 75	6
Plasma	Adams et al. (28)^c^	USA	NA	Arizona, USA	NA	55	44	10.0 ± 3.1	49/6	543 ± 107	μg/L	11.0 ± 3.1	39/5	549 ± 120	7
Serum	Zhu et al. (27) (2)^c^	China	DSM-5	The Maternal and Child Care Health Hospital of Hainan Province, China	The high-performance liquid chromatography method	293	101	4.28 ± 1.37	250/43	296.77 ± 86.17 ^a^	μg/L	4.35 ± 1.18	50/51	297.63 ± 66.60 ^a^	7

a *Data obtained from authors; Age presented as Mean ± SD*.

b *Age range; DSM, the Diagnostic and Statistical Manual of Mental Disorders*.

c *The measuring unit of vitamin A have been converted*.

*NA, not available*.

### Quantitative Data Synthesis

**Figure 2:**
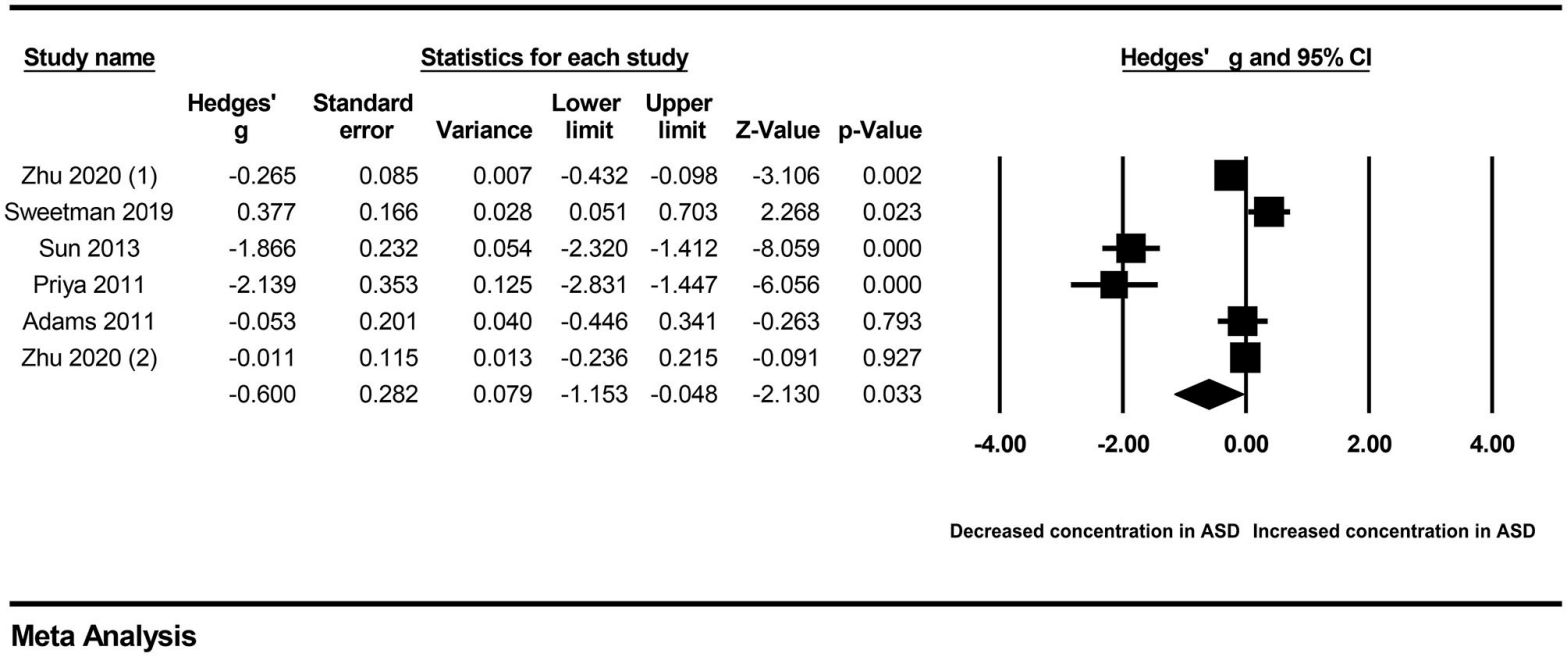
Forest plot for the random-effects meta-analysis.

Because obvious heterogeneity existed across studies (*I*^2^ = 94.838, *P* < 0.001), we performed a random-effects meta-analysis based on the extracted data of six studies encompassing 935 children with ASD and 516 healthy controls. The pooled ES and its corresponding 95% CI were calculated according to the extracted data. The results of the overall comparison showed that vitamin A levels in peripheral blood were significantly decreased in autistic children compared with healthy children (Hedges' g = −0.600, 95% CI: −1.153 to −0.048, *P* = 0.033) (Figure 2).

### Investigation of Heterogeneity

**Figure 3:**
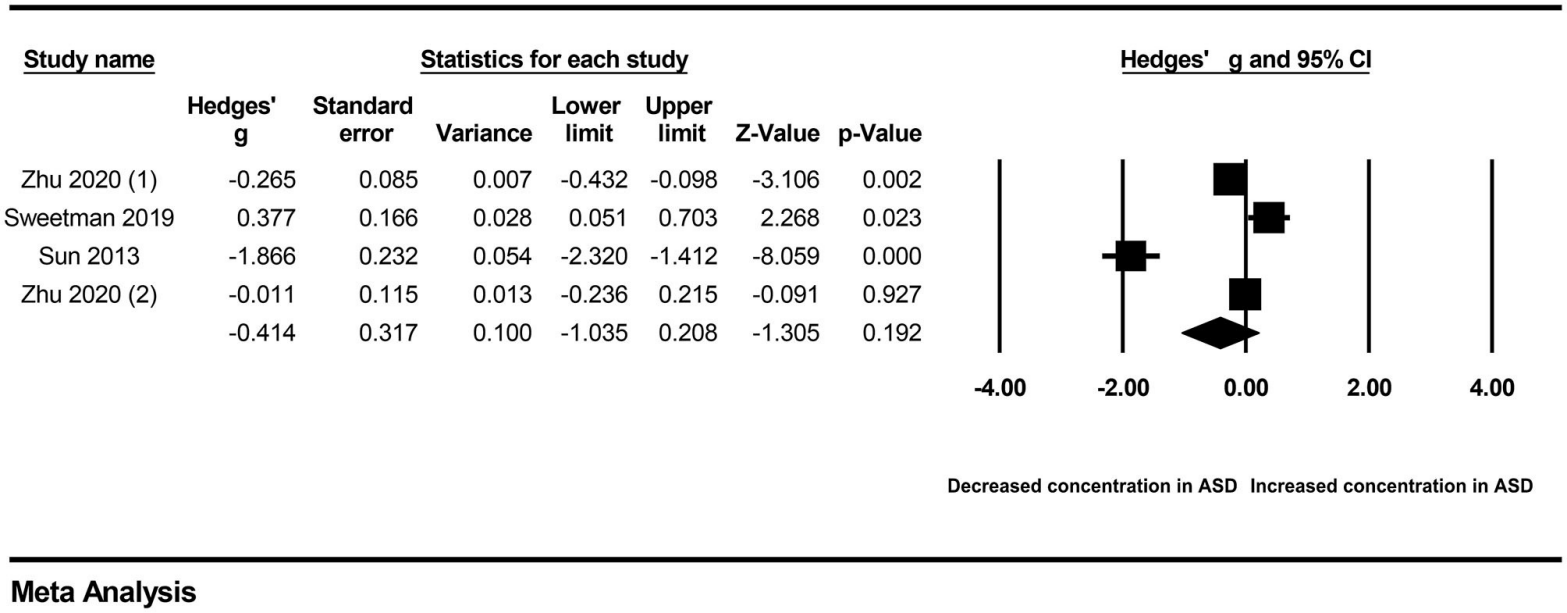
Forest plot for the random-effects meta-analysis of the serum subgroup.

**Figure 4:**
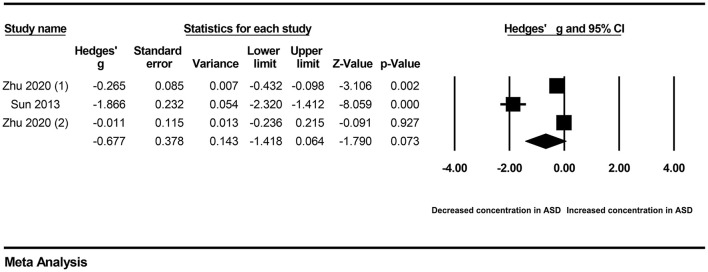
Forest plot for the random-effects meta-analysis of the Chinese subgroup.

First, subgroup analyses were performed based on different sample types and populations. Due to the limitation of the number of included studies, we only explored whether serum samples and Chinese populations could result in significant heterogeneity across studies. The results revealed no significant association between vitamin A levels and ASD in either the serum subgroup (Hedges' g = −0.414, 95% CI: −1.035 to 0.208, *P* = 0.192) (Figure 3) or the Chinese subgroup (Hedges' g = −0.677, 95% CI: −1.148 to 0.064, *P* = 0.073) (Figure 4). Moreover, heterogeneity across studies did not decrease significantly in the serum subgroup (*I*^2^ = 95.524, *P* < 0.001) or Chinese subgroup (*I*^2^ = 96.170, *P* < 0.001). This result suggested that the serum and Chinese subgroups may not play important roles in the generation of heterogeneity in our meta-analysis. Next, a Galbraith plot was employed to assess the effect of studies on heterogeneity in our meta-analysis. Three studies conducted by Sweetman et al. (31), Sun et al. (30) and Priya et al. (29) were significantly displaced from the center of the zero line in the Galbraith plot (Supplementary Figure 1), indicating that these studies may affect the results of heterogeneity analysis. After removing these studies, no significant heterogeneity was found across studies (*I*^2^ = 42.483%, *P* = 0.176). Significantly decreased peripheral vitamin A levels were still found in autistic children compared with healthy children (Hedges' g = −0.162, 95% CI −0.289 to −0.035, *P* = 0.012) (Table 2; Supplementary Figure 2).

**Table 2:** Summary of meta-analysis results.

				**Tests of association**				**Tests of heterogeneity**			**Publication bias**
**Groups**	**Studies (*n*)**	**Case (*n*)**	**Control (*n*)**	**Model**	**Hedges' g (95% CI)**	**Z**	***P*-value**	**Q-value**	***P*-value**	**I^**2**^ (%)**	**Egger's *P*-value**
**Total**	6	935	516	RE	−0.600 (−1.153 to −0.048)	−2.130	0.033	96.871	<0.001	94.838	0.269
**Subgroups**											
Serum	4	865	427	RE	−0.414 (−1.035 to 0.208)	−1.305	0.192	67.029	<0.001	95.524	
Chinese	3	791	355	RE	−0.677 (−1.148 to 0.064)	−1.790	0.073	52.213	<0.001	96.170	
After removing studies	3	793	346	FE	−0.162 (−0.289 to −0.035)	−2.499	0.012	3.477	0.176	42.483	

*RE, random effects model; FE, fixed effects model*.

### Publication Bias

The funnel plot was drawn based on the effect sizes against the precision for each included study. Although the shape of the funnel plot was slightly asymmetrical (Supplementary Figure 3), the result of Egger's test did not reveal obvious publication bias in our meta-analysis (*P* = 0.269).

## Discussion

Our meta-analysis was undertaken to investigate alterations in peripheral vitamin A levels in children with ASD compared to healthy children and found that peripheral vitamin A concentrations were significantly decreased in children with ASD compared to healthy children. These results were consistent with many previous studies (23, 32). Obvious publication bias could have suppressed false-negative results or could have magnified false-positive results. There was no indication that the results of our present meta-analysis were affected by publication bias. Sensitivity analysis was not performed due to the limited number of included studies. We also noted that high levels of heterogeneity across studies existed in the present meta-analysis, which may have influenced on the reliability of the results. Therefore, we conducted subgroup analyses based on serum and Chinese children. However, there was no significant decrease in heterogeneity across studies in the serum subgroup or in the Chinese subgroup. This finding indicated that these factors may not be the source of heterogeneity. Peripheral vitamin A concentrations were not significantly decreased in children with ASD compared to healthy children in subgroup analyses. This result may be attributed to the limited number of included studies. Only three studies were included in each subgroup, which was sufficient for our analysis. Furthermore, a Galbraith plot was drawn to identify the studies resulting in obvious heterogeneity in the present meta-analysis. Three studies obviously deviated from the boundary in the Galbraith plot. After removing these studies, the heterogeneity across studies was significantly reduced, and the levels of vitamin A in the peripheral blood of ASD patients were significantly lower than those of the control group. All of these results indicated the credibility of our results. Therefore, our present results suggested that a significant association potentially exists between peripheral vitamin A levels and ASD.

Our meta-analysis with the largest sample size synthesized data on peripheral vitamin A alterations in ASD and found that children with ASD had decreased peripheral vitamin A levels. The effect of vitamin A on ASD still needs to be further clarified. The active metabolite of vitamin A is retinoic acid (RA), which activates retinoic acid receptors (RARs) and/or retinoid X receptors (RXRs) to regulate gene expression in many cells and tissues (33, 34). In a mouse model, RA directly decreased inhibitory synaptic strength in the brain, resulting in excitation/inhibition imbalance (35). *In vitro*, RA could enhance the expression of many candidate molecules related to synaptic formation, preservation, function and differentiation (36). At present, synaptic plasticity and excitation/inhibition imbalance have a significant impact on the development of ASD (37). On the other hand, food selectivity is reported as a common feeding problem among autistic children (38). Children with ASD may have a food preference that may lead to nutritional insufficiency, such as vitamin A and B12 (39). Clinical studies have shown that appropriate vitamin A supplementation could alleviate social impairment in autistic children (40). Based on these reports, a link between peripheral vitamin A levels and ASD seems to exist. Of course, the clinical significance of decreased peripheral vitamin A levels should be treated dialectically. Whether decreased peripheral vitamin A was a cause of ASD is unclear. More basic studies are required to further verify the causal relationship between peripheral vitamin A and ASD.

Some inherent limitations in our meta-analysis should be noted. First, the sample size was moderate, which restricts the strength and quality of evidence. Despite our subgroup analysis, the source of heterogeneity remains unclear. More samples are required in future studies to verify our results. Second, residual confounding factors are a concern. Some potential confounding factors and methodological and clinical confounding factors, such as age and; sex of included subjects, specimen collection, storage, and detection, were not considered in our present meta-analysis due to the limited information. Whether these moderators could partially account for the current results requires further confirmation. Third, ASD is a group of neurodevelopmental disorders. No follow-up test for peripheral vitamin A levels were performed in the included studies, which led to the lack of dynamic data on peripheral vitamin A. Therefore, more longitudinal clinical studies are still needed to further verify our results. Fourth, we did not evaluate the roles of other retinoids in ASD due to the limited information. Fifth, whether children with ASD suffer vitamin A deficiency could not evaluate for the limited information. More clinical studies should be conducted to further address these questions.

## Conclusions

Our study suggested that decreased vitamin A levels in peripheral blood may be a manifestation of ASD children compared with healthy children, which strengthens the clinical evidence of the abnormal micronutrient profile of autistic children. Studies with larger samples size are needed to further verify the relationship between vitamin A and ASD. The roles of vitamin A in ASD should be clarified in the future.

## Data Availability Statement

The original contributions presented in the study are included in the article/Supplementary Material, further inquiries can be directed to the corresponding author/s.

## Author Contributions

JG conceived the study and corrected the manuscript. JG, NW, and YZ collected the data and drafted the manuscript. All authors contributed to the article and approved the submitted version.

## Funding

This study was supported by the National Nature Science Foundation of China (No. 81701135).

## Conflict of Interest

The authors declare that the research was conducted in the absence of any commercial or financial relationships that could be construed as a potential conflict of interest.

## Publisher's Note

All claims expressed in this article are solely those of the authors and do not necessarily represent those of their affiliated organizations, or those of the publisher, the editors and the reviewers. Any product that may be evaluated in this article, or claim that may be made by its manufacturer, is not guaranteed or endorsed by the publisher.
