# Supplementary material for: Association Between Peripheral Blood Levels of Vitamin A and Autism Spectrum Disorder in Children: A Meta-Analysis
Source: Front Psychiatry. 2021 Sep 30;12:742937. doi: 10.3389/fpsyt.2021.742937 (PMC8515042; doi:10.3389/fpsyt.2021.742937)

Supplementary Figure 1. Galbraith plot for the random-effects meta-analysis.


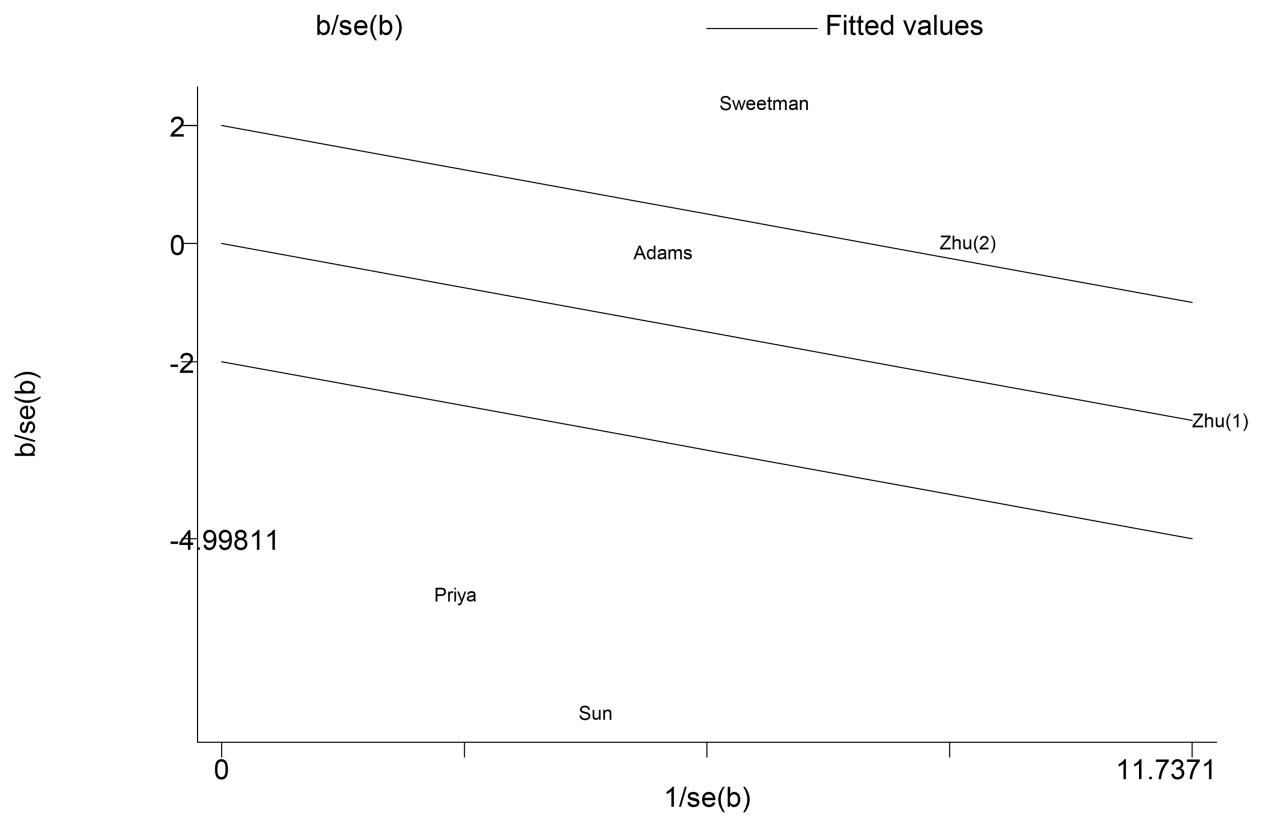


Supplementary Figure 2. Forest plot for the fixed-effects meta-analysis after removing the studies outside the boundaries of the Galbraith plot.


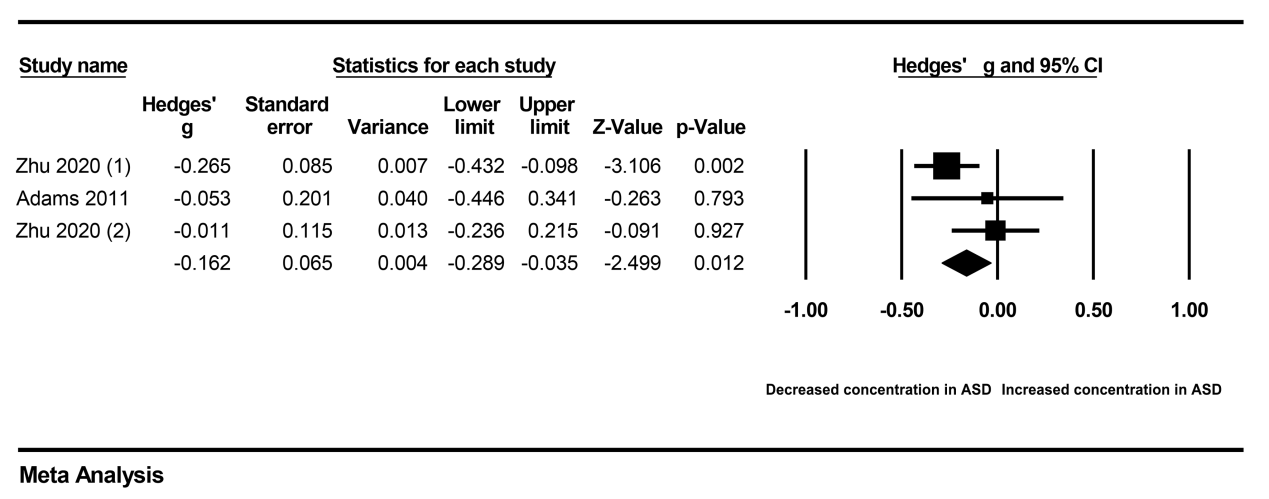


Supplementary Figure 3. Funnel plot of precision using Hedges’ g statistics.


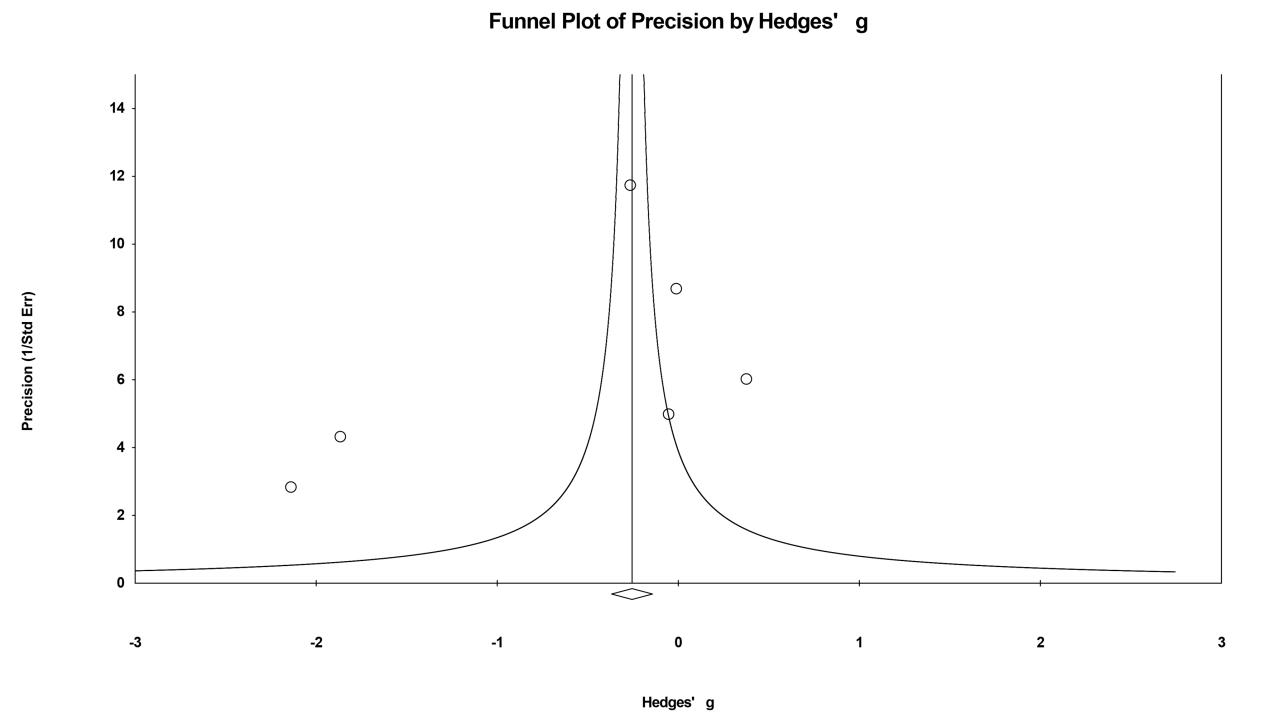

Supplement: Supplementary file 1 [file Data_Sheet_1.DOCX]
